# Effects of exogenous Strigolactone on the physiological and ecological characteristics of *Pennisetum purpureum* Schum. Seedlings under drought stress

**DOI:** 10.1186/s12870-022-03978-y

**Published:** 2022-12-12

**Authors:** Yan Li, Sutao Li, Qixian Feng, Juan Zhang, Xuelin Han, Lei Zhang, Fulin Yang, Jing Zhou

**Affiliations:** 1 College of Animal Sciences (College of Bee Science), Fujian Agriculture and Forestry University, Fuzhou, 350002 China; 2 National Engineering Research Center of Juncao Technology, Fujian Agriculture and Forestry University, Fuzhou, 350002 China

**Keywords:** Strigolactone, Drought stress, Photosynthesis characteristics, Growth performance, Endogenous ABA, *Pennisetum purpureum* Schum

## Abstract

**Background:**

Drought is one of the main environmental factors limiting plant growth and development. *Pennisetum purpureum* Schum. was used to explore the mitigation effects of exogenous strigolactone (SL) on drought stress during the seedling stage. The effects of different concentrations (1, 3, 5, and 7 μmol·L^− 1^) of SL on the photosynthesis characteristics, growth performance, and endogenous abscisic acid (ABA) of *P. purpureum* under drought stress were studied.

**Results:**

Exogenous SL could effectively alleviate the inhibitory effect of drought stress on *P. purpureum* growth. Compared with drought stress, the net photosynthesis rate, stomatal conductance, transpiration rate, and water-use efficiency of the leaves of *P. purpureum* after SL treatment significantly increased, thereby exerting a significant mitigation effect on the decrease in photosystem II maximum photochemical efficiency and the performance index based on light absorption caused by drought. Moreover, the exogenous application of SL can effectively increase the fresh and dry weight of the leaves and roots and the main-root length. After applying SL for 120 h, the ABA content of *P. purpureum* decreased significantly. The activity of key enzymes of photosynthesis significantly increased after 48 h of external application of SL to *P. purpureum*.

**Conclusions:**

SL treatment can improve the photosynthesis performance of *P. purpureum* leaves under drought conditions and increase the antioxidant capacity of the leaves, thereby reducing the adverse effects of drought, promoting the growth of *P. purpureum*, and effectively improving the drought resistance of *P. purpureum*.

## Background

Drought is one of the most important environmental factors affecting plant growth and development [1]. With the looming global climate change, the frequency and intensity of regional droughts are also increasing yearly [2]. On one hand, drought can reduce the photosynthesis rate of leaves and destroy plant photosynthesis organs, in turn leading to the inability of plants to effectively utilize absorbed light energy [3, 4]. On the other hand, drought can affect nutrient acquisition, transport, distribution, and storage, leading to decreased plant-biomass accumulation and root vigor [5, 6]. Plant hormones play a crucial role in regulating plant responses to abiotic stresses [7]. Many phytohormones including abscisic acid (ABA), ethylene (ET), salicylic acid (SA), and jasmonic acid (JA) are involved in the regulation of plant stomata and inducing the expression of genes related to stomatal aperture [7]. Hormones such as ABA, SA, and JA also play important roles in improving chlorophyll (Chl) content, relative water content, and proline content, as well as in increasing the fresh and dry weight of roots and above-ground parts [8]. These hormones are also involved in the scavenging of reactive oxygen species (ROS), maintaining the redox status of plants under stress conditions, and enhancing antioxidant defense responses [9]. Thus, phytohormones play an important role in regulating plant response to abiotic stresses.

Strigolactones (SLs), a class of hormones produced with carotenoids as precursors, affect plant growth and development by regulating various hormone-response pathways of plants and their growth to improve their adaptation to adverse stresses [10, 11]. SLs further reportedly inhibit plant branching, control shape root morphology, enhance primary root cell growth to inhibit adventitious root development and growth, and regulate plant secondary growth [12, 13]. They are also associated with drought resistance, cold tolerance, salt tolerance and other stress tolerance [14–17], and photomorphogenesis [18] and serve as positive regulators of plant responses to certain abiotic stresses [19]. Sattar et al. [20] applied SL to maize (*Zea mays* L.) under drought stress and found that SL improves the water relationship of maize seedlings, increases photosynthesis pigments and gas-exchange parameters, and improves antioxidant enzyme activity. Thus, the tolerance of maize seedlings to drought stress is enhanced. Ha et al. [21] found that *Arabidopsis thaliana* plants deficient in SL biosynthesis and signal transduction exhibit hypersensitivity to drought and salt stress. Treatment with exogenous SL restores the drought-sensitive phenotype of SL-deficient mutants, but not SL-responsive mutants, and enhances drought tolerance in wild-type (WT) plants. The role of SLs as a positive regulator in stress response has been confirmed. Min et al. [22] studied the foliar application of SL to alleviate drought stress in grapes (*Vitis vinifera* L.). They found that GR24 treatment increases the Chl content and Pn of plants and reduces the electrolyte leakage, ROS content, indole acetic acid and zeatin riboside contents in the roots and leaves under drought stress. Consequently, the adverse effects of drought are ameliorated.

Photosynthesis is one of the most important physiological processes that are inhibited in plants under drought stress. Studies have shown that SL can inhibit the activity of Chl-degrading enzymes, regulate the binding of Chl to membrane proteins, maintain the stability of the chloroplast thylakoid membrane, and continuously enhance the photosynthesis capacity [23]. SL can also increase the stomatal conductance (Gs), transpiration rate (Tr), and intercellular CO_2_ concentration (Ci) of plants under stress conditions, thereby increasing the Pn [16]. SL can ease the photosynthesis process as well by maintaining the stability of the photosystem II (PSII) supercomplex, increasing D1 protein turnover, the photosynthetic electron transport and the demand for ATP and NADPH in the Calvin cycle, and the efficiency of the photosystem, ultimately promoting plant growth and development [24–26]. Photosynthesis in higher plants can be classified as C_3_, C_4_, and crassulacean acid metabolism according to the way they fix carbon during the production of different initial photosynthesis products [27]. Among the many enzymes involved in the C_4_ photosynthesis pathway, phosphoenolpyruvate carboxylase (PEPC), pyruvate phosphate dikinase (PPDK), and NADP-malic enzyme (NADP-ME) are considered to be the most important [28–30]. The average Pn of rice (*Oryza sativa* L.) transduced with C_4_ model cereal (*Setaria italica* L.) PPDK and NADP-ME genes increases by 18 and 12%, respectively, and is positively correlated with increased photosynthesis pigment content [31]. Transgenic rice plants transfer more absorbed light energy to photochemical reactions than WT plants, and transgenic plants exhibit increased yield as evidenced by increased plant height, spike length, spike weight, and thousand grain weight [31]. This finding indicates that the activity of key photosynthesis enzymes is important for photosynthesis and plant yield.

*Pennisetum purpureum* Schum. is a perennial C_4_ plant with tall plants, well-developed root systems, strong tillering capacity, and huge biomass [32]. *P. purpureum* has few pests and diseases, fast growth, and high yield. It contains a large amount of crude protein and soluble sugar, which is a kind of high-yielding and high-quality forage grass with economic and ecological benefits for sustainable development. At present, studies on *P. purpureum* have focused on application and nutritional value [32–34], whereas the effects of plant growth regulators such as SL on drought stress in *P. purpureum* have not been reported. Accordingly, the present study was conducted to determine the effects of exogenous SL on the growth, photosynthesis characteristics, fresh weight, dry weight, main root length, endogenous abscisic acid (ABA), and key photosynthesis enzyme activities of *P. purpureum* under drought stress. We aimed to reveal the regulatory mechanism of exogenous SL application on *P. purpureum* in response to drought stress and provide a theoretical basis for the application and research of exogenous plant growth regulators such as SLs and thus improve the drought resistance of *P. purpureum*.

## Results

### Effect of SL on the photosynthesis parameters of leaves of *P. purpureum* under drought stress

Table 1 shows the net Pn and Tr of *P. purpureum* leaves, which continuously decreased with the severity of drought. Gs, Ci, and WUE showed a decreasing, increasing, and decreasing trend. These results all significantly differed from those of the normally watered control (*P* < 0.05). Under drought stress, the foliar application of SL to *P. purpureum* effectively alleviated the inhibitory effect caused by drought stress. After 24 h of hormone spraying, the Pn and Gs of the T1, T2, and T3 treatment groups were significantly elevated, with significant differences (*P* < 0.05) from the drought-treatment group (D group). The most significant relief effect was observed in T2, which was close to that of the normal watering group. The Tr of all hormone-treatment groups was significantly higher than that of the D group, with the most significant effect on T2 and T3. The Ci of all hormone-treatment groups was significantly higher than that of the D group. WUE significantly improved only in T1, which significantly differed from that of the D group (*P* < 0.05).

**Table 1:** Effect of SL on the photosynthetic parameters of *P. purpureum* leaves under drought stress

Treatment		Net photosynthetic rate	Stomatal conductance	Transpiration rate	Intercellular CO_2_ concentration	Water use efficiency
		μmol·(m^2^·s)^−1^	μmol·(m^2^·s)^− 1^	mmol·(m^2^·s)^− 1^	μmol∙mol^− 1^	mmol·mol^− 1^
24 h	N	19.94 ± 0.24^a^	174.20 ± 1.80^a^	4.46 ± 0.02^a^	154.00 ± 2.28^b^	4.47 ± 0.03^a^
	D	12.14 ± 0.51^c^	98.00 ± 2.55^b^	3.23 ± 0.07^c^	148.80 ± 8.48^b^	3.76 ± 0.16^b^
	T1	16.54 ± 0.90^b^	178.60 ± 8.27^a^	3.81 ± 0.11^b^	189.60 ± 10.12^a^	4.35 ± 0.21^a^
	T2	17.06 ± 1.73^ab^	184.20 ± 23.50^a^	4.43 ± 0.34^a^	184.60 ± 3.94^a^	3.81 ± 0.11^b^
	T3	15.94 ± 1.01^b^	172.80 ± 11.68^a^	4.54 ± 0.15^a^	189.60 ± 2.73^a^	3.50 ± 0.15^bc^
	T4	12.42 ± 1.08^c^	123.80 ± 10.88^b^	4.00 ± 0.17^ab^	183.20 ± 5.68^a^	3.08 ± 0.15^c^
48 h	N	18.80 ± 0.33^a^	137.40 ± 4.20^b^	4.21 ± 0.06^a^	133.20 ± 6.35^c^	4.47 ± 0.14^a^
	D	11.14 ± 0.56^bc^	103.40 ± 6.71^c^	2.66 ± 0.09^cd^	185.20 ± 15.86^b^	4.21 ± 0.25^ab^
	T1	12.48 ± 1.00^b^	165.60 ± 16.34^a^	3.91 ± 0.20^ab^	225.80 ± 8.65^a^	3.19 ± 0.19^c^
	T2	12.84 ± 0.82^b^	133.60 ± 9.50^b^	3.56 ± 0.17^b^	194.00 ± 7.12^b^	3.61 ± 0.18^bc^
	T3	11.94 ± 0.30^b^	112.00 ± 4.44^bc^	2.85 ± 0.07^c^	181.40 ± 5.07^b^	4.19 ± 0.11^ab^
	T4	9.38 ± 0.97^c^	94.00 ± 6.56^c^	2.38 ± 0.12^d^	201.20 ± 10.20^ab^	3.90 ± 0.26^ab^
120 h	N	19.56 ± 0.54^a^	168.60 ± 16.50^a^	5.04 ± 0.31^a^	138.60 ± 7.26^b^	4.24 ± 0.20^bc^
	D	7.66 ± 1.15^d^	59.20 ± 8.11^d^	1.78 ± 0.19^c^	163.60 ± 8.43^a^	3.91 ± 0.16^c^
	T1	14.98 ± 0.50^b^	128.20 ± 5.28^b^	3.16 ± 0.15^b^	159.20 ± 1.53^a^	4.76 ± 0.14^ab^
	T2	13.16 ± 0.75^b^	99.00 ± 8.45^c^	2.76 ± 0.10^b^	137.40 ± 6.55^b^	4.75 ± 0.16^ab^
	T3	10.42 ± 0.93^c^	76.00 ± 8.23^cd^	2.11 ± 0.18^c^	138.60 ± 11.05^b^	4.96 ± 0.25^a^
	T4	7.50 ± 1.02^d^	53.00 ± 5.84^d^	1.65 ± 0.15^c^	148.20 ± 11.36^ab^	4.46 ± 0.26^abc^

Different lowercase letters in the same column indicate significant differences (*P* < 0.05). N: normal watering treatment; D: drought stress treatment only; T1, T2, T3, and T4 represent the different treatment concentrations of SL under drought stress (1, 3, 5, and 7 μmol·L^− 1^, respectively)

After 48 h of hormone spraying, the Pn, Gs, and Tr in T1, T2, and T3 were higher than those in the D group, whereas the Pn, Gs, and Tr in T4 were lower than those in the D group. Ci most significantly increased in T1, and WUE showed an inhibitory trend compared with the D group in all three groups except T3. This finding indicated that the relief effect of hormone on Tr was higher than that on Pn. After 120 h of hormone spraying, the Pn, Gs, Tr, and WUE were significantly higher in T1, T2, and T3 than those in the D group. Overall, T1 showed the most significant relief effect, and Ci showed a decreasing trend in all treatment groups.

### Effect of SL on the physiological properties of Chl fluorescence in leaves of *P. purpureum* under drought stress

**Fig. 1:**
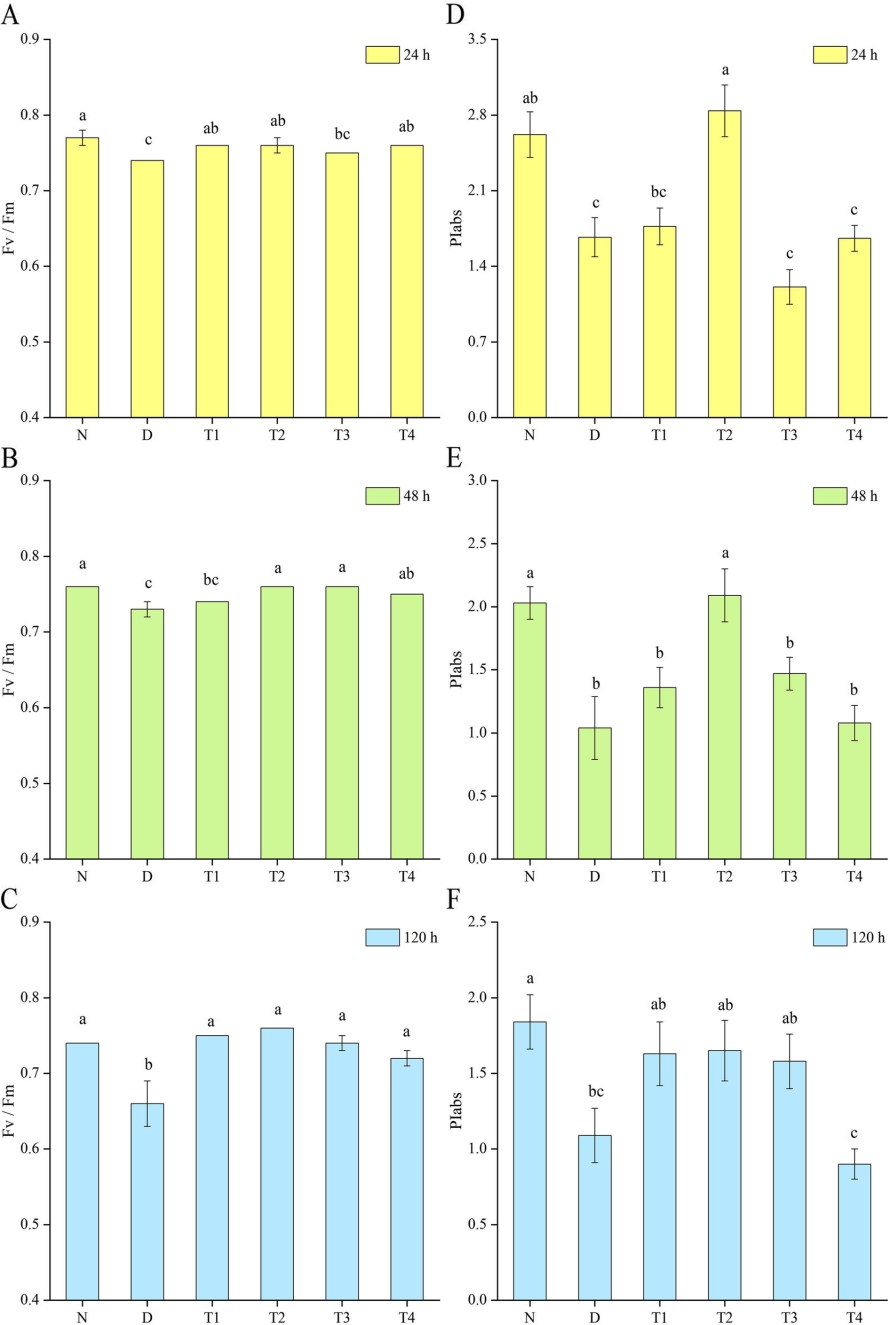
Effect of spraying SL on the physiological characteristics of Chl fluorescence of *P. purpureum* leaves under drought stress. Effects of SL on Fv/Fm (**a**, **b**, and **c**) and PIabs (**d**, **e**, and **f**) of *P. purpureum* leaves. Different lowercase letters at the same time indicate significant differences (*P* < 0.05). N: normal watering treatment; D: drought stress treatment only; T1, T2, T3, and T4 represent the different treatment concentrations of SL under drought stress (1, 3, 5, and 7 μmol·L^− 1^ respectively)

Figure 1 shows that the maximum photochemical efficiency of PSII (Fv/Fm) and the performance index based on the absorption of light energy (PIabs) of the leaves of *P. purpureum* under drought stress were significantly lower than those of the normal control. This finding indicated that the photosynthesis performance of *P. purpureum* was severely affected by photoinhibition under drought stress, and the photoinhibition became more serious with the severity of drought. The photoinhibition that occurred in PSII of *P. purpureum* was significantly alleviated by spraying SL on its leaves, and Fv/Fm and PIabs were significantly enhanced. After 24 h of hormone spraying, T1, T2, T3, and T4 showed significant alleviating effects compared with the D group, and the Fv/Fm values of T1, T2, and T4 were significantly higher than that in the D group. After 48 h of hormone spraying, T1, T2, T3, and T4 differed significantly (*P* < 0.05) compared with the D group. The Fv/Fm values of T2, T3, and T4 were significantly higher than those of the D group. After 24 and 48 h of hormone spraying, the PIabs of T2 differed significantly (*P* < 0.05) from that of the D group and reached the level of the normal control group. Therefore, the relief effect of T2 was the most significant. After 120 h of hormone spraying, the relief effect of T1, T2, T3, and T4 was significantly higher than that of the D group. The Fv/Fm values of each treatment group were significantly higher than that of the D group, and the relief effect of PIabs was the most obvious in T2.

### Effect of SL on *P. purpureum* biomass under drought stress

As shown in Table 2, drought stress had a significant effect on the biomass of *P. purpureum*, and its leaf fresh weight and dry weight, root fresh weight and dry weight, and leaf and root water content were significantly lower than those of the normal watering-treatment group. The application of exogenous SL to *P. purpureum* under drought stress alleviated the accumulation of biomass in *P. purpureum*, but it was related to the SL concentration. After 24 h of hormone spraying, the relief effect was most significant for leaf fresh weight and dry weight in T2 and T3, for root fresh weight and dry weight in T1, and for leaf and root water content in T2. After 48 h of hormone spraying, the leaf fresh weight, dry weight, and leaf water content were most significantly relieved by T1, followed by T2. The root fresh weight and dry weight were most significantly relieved by T1 and T2. No significant differences were observed in root water content at this time. After 120 h of hormone spraying, the leaf fresh weight, dry weight, and leaf water content were most significantly relieved by T1, the root fresh weight and dry weight were most significantly relieved by T3, and the root water content was most significantly relieved by T2. Therefore, T1 and T2 had the most significant effect on the biomass of *P. purpureum* under drought stress.

**Table 2:** Effect of SL on the biomass of *P. purpureum* under drought stress

Treatment		Leaf fresh weight (g·plant^− 1^)	Leaf dry weight (g·plant^− 1^)	Leaf water content (%)	Root fresh weight (g·plant^− 1^)	Root dry weight (g·plant^− 1^)	Root water content (%)
24 h	N	6.32 ± 0.93^a^	1.45 ± 0.33^a^	77.43 ± 2.18^a^	11.80 ± 1.47^a^	1.53 ± 0.25^a^	86.99 ± 0.06^a^
	D	3.60 ± 0.35^b^	0.93 ± 0.09^ab^	73.79 ± 2.57^a^	6.07 ± 0.34^b^	1.00 ± 0.08^b^	83.55 ± 0.52^b^
	T1	3.28 ± 0.12^b^	0.83 ± 0.07^b^	74.70 ± 1.20^a^	7.38 ± 1.18^b^	1.08 ± 0.21^ab^	85.33 ± 0.89^ab^
	T2	3.90 ± 0.42^b^	0.87 ± 0.15^b^	77.78 ± 1.36^a^	5.85 ± 0.78^b^	0.85 ± 0.15^b^	85.51 ± 0.89^ab^
	T3	3.90 ± 0.39^b^	1.02 ± 0.12^ab^	73.98 ± 1.30^a^	6.37 ± 0.62^b^	0.95 ± 0.09^b^	85.02 ± 0.87^ab^
	T4	3.25 ± 0.12^b^	0.78 ± 0.04^b^	75.86 ± 1.38^a^	5.22 ± 0.49^b^	0.77 ± 0.09^b^	85.37 ± 0.43^ab^
48 h	N	6.73 ± 0.86^a^	1.38 ± 0.25^a^	79.59 ± 0.44^a^	11.83 ± 2.08^a^	1.43 ± 0.23^a^	87.75 ± 0.82^a^
	D	3.12 ± 0.42^b^	0.78 ± 0.16^b^	75.21 ± 1.89^a^	6.27 ± 1.40^ab^	0.98 ± 0.09^a^	84.65 ± 0.95^ab^
	T1	5.37 ± 0.97^ab^	1.03 ± 0.18^ab^	80.47 ± 1.95^a^	7.07 ± 1.43^ab^	1.10 ± 0.20^a^	84.27 ± 1.04^ab^
	T2	4.42 ± 0.28^ab^	0.97 ± 0.02^ab^	77.91 ± 1.68^a^	8.30 ± 1.47^ab^	1.55 ± 0.58^a^	82.27 ± 1.09^b^
	T3	4.12 ± 1.18^ab^	0.95 ± 0.22^ab^	75.55 ± 1.02^a^	5.73 ± 0.62^b^	1.02 ± 0.37^a^	81.73 ± 1.65^b^
	T4	3.77 ± 0.49^b^	0.92 ± 0.12^ab^	75.59 ± 1.05^a^	5.42 ± 1.05^b^	0.83 ± 0.11^a^	84.27 ± 0.92^ab^
120 h	N	7.68 ± 0.85^a^	1.45 ± 0.18^a^	81.13 ± 0.57^a^	12.88 ± 0.35^a^	1.70 ± 0.11^a^	86.55 ± 0.89^a^
	D	3.20 ± 0.06^b^	0.73 ± 0.09^b^	76.97 ± 0.93^b^	7.20 ± 0.60^b^	1.17 ± 0.06^bc^	83.70 ± 0.60^a^
	T1	4.42 ± 0.47^b^	1.02 ± 0.13^ab^	77.04 ± 1.00^b^	5.85 ± 0.69^b^	0.88 ± 0.02^bc^	84.37 ± 1.90^a^
	T2	4.07 ± 0.91^b^	1.00 ± 0.12^ab^	75.87 ± 1.54^b^	6.42 ± 0.19^b^	0.87 ± 0.21^bc^	86.62 ± 0.80^a^
	T3	4.35 ± 0.35^b^	1.05 ± 0.14^ab^	76.05 ± 1.72^b^	9.02 ± 1.56^b^	1.35 ± 0.24^ab^	85.03 ± 0.57^a^
	T4	4.20 ± 1.05^b^	1.07 ± 0.09^ab^	71.52 ± 3.57^b^	5.83 ± 0.46^b^	0.80 ± 0.06^c^	86.26 ± 0.47^a^

Different lowercase letters in the same column indicate significant differences (*P* < 0.05). N: normal watering treatment; D: drought stress treatment only; T1, T2, T3, and T4 represent the different treatment concentrations of SL under drought stress (1, 3, 5, and 7 μmol·L^−1^, respectively)

### Effect of SL on the root length of *P. purpureum* under drought stress

**Fig. 2:**
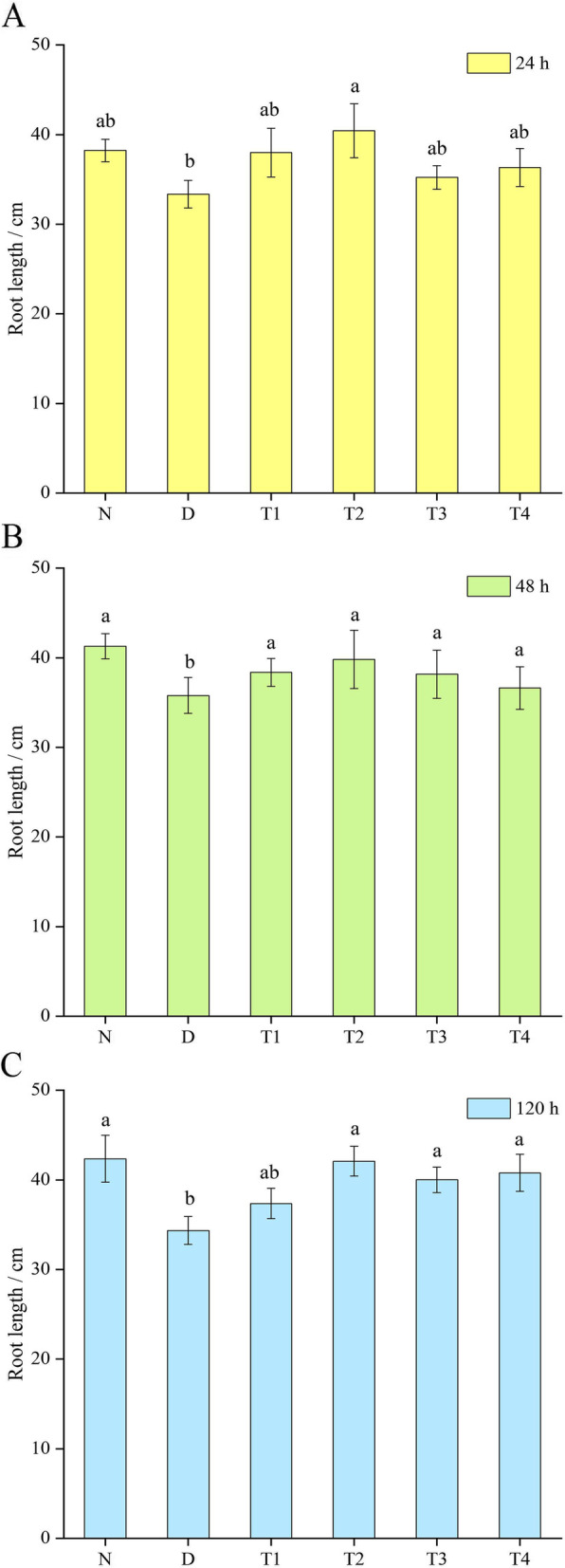
Effect of spraying SL on the root length of *P. purpureum* under drought stress. Effects of SL on root length (**a**, **b**, and **c**) of *P. purpureum*. Different lowercase letters at the same time indicate significant differences (*P* < 0.05). N: normal watering treatment; D: drought stress treatment only; T1, T2, T3, and T4 represent the different treatment concentrations of SL under drought stress (1, 3, 5, and 7 μmol·L^− 1^ respectively)

As shown in Fig. 2, the root length of the D group was significantly lower than that of the normal treatment group. After spraying SL, their root lengths significantly improved and differed significantly from those of the D group (*P* < 0.05). The most significant effects on the root system were observed in T2 at 24, 48, and 120 h after hormone spraying. The effect of T1 on the root system was better than that of T3 and T4 in the first 48 h. The effect of T3 and T4 on the root system was better than that of T1 after 120 h.

### Effect of SL on the ABA content of *P. purpureum* under drought stress

**Fig. 3:**
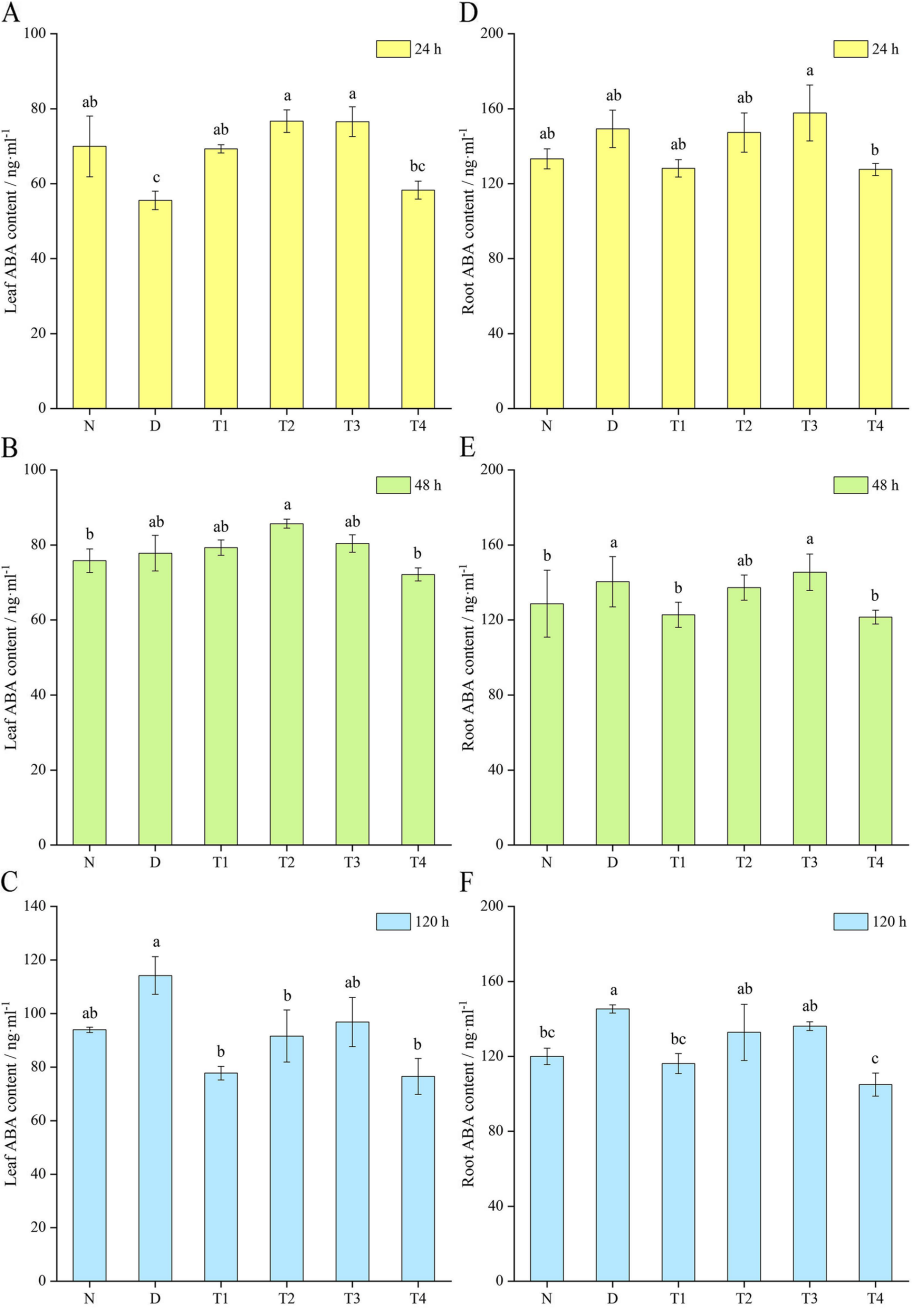
Effect of spraying SL on the ABA content of *P. purpureum* under drought stress. Effects of SL on leaf ABA content (**a**, **b**, and **c**) and root ABA content (**d**, **e**, and **f**) of *P. purpureum*. Different lowercase letters at the same time indicate significant differences (*P* < 0.05). N: normal watering treatment; D: drought stress treatment only; T1, T2, T3, and T4 represent the different treatment concentrations of SL under drought stress (1, 3, 5, and 7 μmol·L^− 1^ respectively)

Figure 3 shows that with prolonged drought time, the ABA content in *P. purpureum* leaves showed a continuous increasing trend, and that in *P. purpureum* roots showed an increasing, decreasing, and increasing trend. The ABA content of *P. purpureum* leaves under drought stress was initially lower and then became higher than that of the normal control group. Meanwhile, the ABA content of *P. purpureum* roots under drought stress was always higher than that of the normal control group. After spraying SL on *P. purpureum* under drought stress, the ABA content of leaves showed a continuous increase in all treatment groups, and the ABA content of the roots showed a continuous decrease. However, the ABA content in roots was always higher than that in leaves. After 24 h of hormone spraying, the ABA content in the leaves of all treatment groups was higher than that of the D group. The ABA content in the roots of T3 was higher than that of the D group, and the ABA content in the roots of the remaining three groups was lower than that of the D group. After 48 h of hormone spraying, the ABA content in the leaves of T1, T2, and T3 was higher than that of the D group. The ABA content in the leaves of T4 was lower than that of the D group. The ABA content in the roots of T3 was higher than that of the D group. The ABA content in the roots of T1, T2, and T4 was lower than that of the D group. After 120 h of hormone spraying, the ABA content in the leaves and roots of the treatment groups (all concentrations) was lower than that of the D group.

### Effect of SL on key photosynthesis enzyme activities in *P. purpureum* under drought stress

**Fig. 4:**
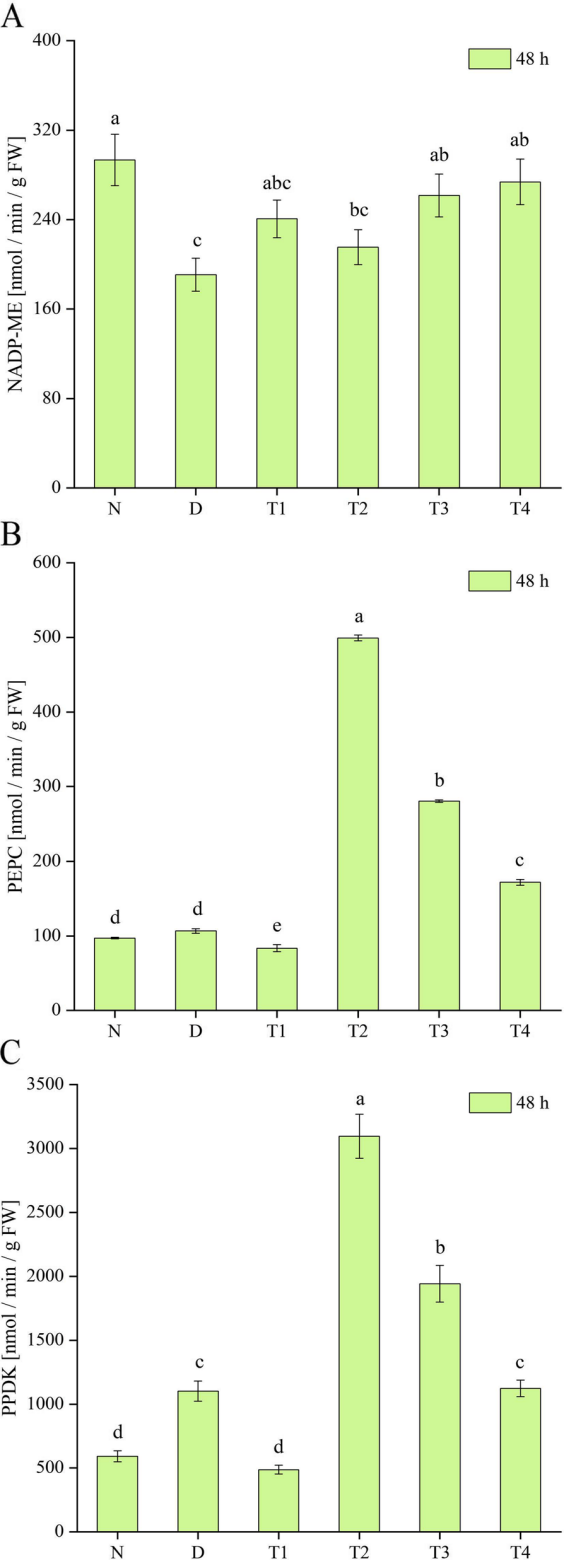
Effect of spraying SL on the activities of key enzymes in the photosynthesis of *P. purpureum* under drought stress. Effects of SL on NADP-ME (**a**), PEPC (**b**), and PPDK (**c**) activities of *P. purpureum*. Different lowercase letters at the same time indicate significant differences (*P* < 0.05). N: normal watering treatment; D: drought stress treatment only; T1, T2, T3, and T4 represent the different treatment concentrations of SL under drought stress (1, 3, 5, and 7 μmol·L^− 1^ respectively)

As shown in Fig. 4, the NADP-ME activity in the D group was significantly lower than that in the normal-watering group, the PEPC and PPDK activities were higher than those in the normal-watering group, and the PPDK activity differed significantly (*P* < 0.05) from that of the normal-watering group. The NADP-ME, PEPC, and PPDK activities were significantly higher in T2 and T3 after spraying SL onto *P. purpureum* under drought stress. The NADP-ME activity in T1 was higher than that in the D group, and the PEPC and PPDK activities were lower than those in the D group. The NADP-ME and PEPC activities were significantly higher in T4 than in the D group, and the PPDK activity did not change significantly compared with the D group.

## Discussion

Drought is one of the major abiotic stress factors affecting plant growth. To cope with drought stress, plants have developed various self-protection and self-defense mechanisms during their long-term evolution [35, 36]. Plants respond to drought by regulating morphological and physiological characteristics such as root growth, leaf structure, stomatal movement, and photosynthesis. However, the ability of plants to adapt to drought by altering their own physiological and biochemical metabolism is limited and has significant effects only in the early stages of drought; with the severity of drought, the plant’s own metabolism becomes disturbed, eventually affecting plant growth and morphogenesis, resulting in reduced biomass accumulation [37, 38]. The results of this experimental study showed that under drought stress, the fresh weight, dry weight, and water content of the leaves and roots of *P. purpureum* were significantly lower than those of the normal-watering group. After foliar spraying of SL onto *P. purpureum* under drought stress, its dry and fresh weight and water content increased, and the growth inhibition caused by drought stress was alleviated, with the best alleviation effect observed in T1 and T2. This finding indicated that the application of SL promoted the growth of *P. purpureum* under drought conditions, which may be related to phytohormone interactions. More studies have shown that SL can interact with growth hormones and cytokinins to regulate plant biomass accumulation and promote plant growth and development [39, 40]. Sattar et al. [20] also showed that exogenous application of SL increases the dry weight and relative water content of maize in above- and below-ground under drought stress.

Drought stress causes the stomatal closure of plant leaves, causing a decrease in leaf Gs, which in turn reduces Tr to reduce water loss. However, stomatal closure reduces water loss while also reducing CO_2_ entry, decreasing the CO_2_ concentration in the leaves and leading to a continuous decrease in Pn, thereby inflicting more serious damage to plants with increased drought severity [41, 42]. In this experiment, drought stress caused a significant decrease in net Pn, Gs, Tr, and WUE in *P. purpureum* leaves, and Ci initially decreased and then increased. The inhibition of photosynthesis and reduction of Pn caused by drought stress can be attributed to stomatal and nonstomatal factors. If Gs and Ci decreased, the net Pn was primarily affected by stomatal factors. If Gs decreased but Ci increased, the net Pn was affected mostly by nonstomatal factors, indicating that photosynthesis was limited by stomatal and nonstomatal factors in this experiment [43–45]. The net Pn, Gs, Tr, and WUE of *P. purpureum* leaves were enhanced by the exogenous application of SL. Particularly, T1 and T2 had the most significant enhancement effect, whereas T4 had an insignificant alleviation effect, indicating that the low concentration of SL could alleviate the inhibitory effect of drought stress on *P. purpureum*, and enhance its photosynthesis capacity. This finding may be due to the fact that SL can regulate the binding of Chl to membrane proteins and thus maintain the stability of chloroplast cyst-like membranes, thereby enhancing the plant’s ability to use light energy to overcome photosystem damage and the photosynthesis performance of the plant [17]. Sedaghat et al. [46] obtained similar findings in their study on the effect of SL on wheat (*Triticum aestivum* L.) under drought stress, in which SL application enhances the net Pn, Gs, and Tr of wheat. Thula et al. [47] showed that the effect on photosynthesis is primarily attributed to SL compared with other plant hormones. In the present study, SL had a more pronounced effect on stomatal conductance. Stomatal conductance and transpiration rates were significantly higher in TI and T2 than in the D group at 24, 48, and 120 h. The effect of SL on stomatal conductance and transpiration rate was higher in T2 than in the D group. The opening of stomata led to increased transpiration rate and also promoted the entry of atmospheric CO_2_, which in turn enhanced the photosynthesis rate [48]. This phenomenon could be a strategy of SL to alleviate drought stress by opening stomata and thus maintain the CO_2_ supply and enhance photosynthesis performance. Stomatal conductance may have also been influenced by osmoregulation and soluble sugar and proline contents may have been elevated by SL treatment. Consequently, the water-retention capacity of the plant and the water-uptake capacity of the plant roots were enhanced, thereby maintaining the water balance in the plant and thus opening the stomata [46, 49].

Drought stress inflicts substantial damage to the PSII reaction center in plants, and the damage to PSII accelerates Chl degradation and exacerbates the denaturation and inactivation of complex proteins in the PSII structure. These phenomena increase the photoinhibition and significantly reduce the light-energy-conversion efficiency of plants [50–52]. Chl fluorescence parameters can reflect the absorption and conversion of light energy in plant leaves under drought stress in terms of light energy conversion and electron-transfer efficiency of leaf PSII, which is an important basis for determining the degree of photosystem damage under drought stress conditions [53, 54]. Among them, Fv/Fm is the maximum photochemical efficiency of PSII, which can reflect the maximum capacity of photosystem reaction centers to utilize light energy, whereas PIabs reflects the overall performance of PSII [55–57]. In the present study, drought stress led to a significant decrease in Fv/Fm and PIabs in *P. purpureum* leaves, which continuously decreased with prolonged stress time. This finding indicated that the PSII reaction center of *P. purpureum* leaves was damaged by stress, and the damage became more serious with drought severity. Fv/Fm and PIabs were significantly elevated after the exogenous application of SL, indicating that SL effectively improved the function of PSII and alleviated photoinhibition. This finding may be due to the fact that SL alleviates the photosynthesis process by maintaining the stability of PSII or increasing the turnover of D1 protein and by improving the photosynthetic electron transport and the demand for NADPH in the Calvin cycle. Min et al. [22] obtained similar results in a study on grapes, where spraying of SL under drought stress elevated Fv/Fm levels. However, in the present study, the alleviating effect of SL on drought stress showed concentration dependence. Fv/Fm and PIabs had the best alleviating effect in T2 at all three sampling time points, and the difference was significant (*P* < 0.05) compared with the D group. Conversely, T4 produced a mitigating effect only on Fv/Fm, with almost no effect on PIabs, and PIabs was lower than those of the D group at 120 h. The high concentration may have slightly inhibited some of the PSII activity. Therefore, selecting the appropriate concentration in the application of exogenously administered plant hormones is important to relieve environmental stress.

The root system is the main organ for water and nutrient absorption in plants, and drought usually leads to self-thinning of the root system, resulting in decreased root-absorption capacity and the death of some roots by abscission, seriously affecting normal plant growth [58, 59]. In the present experiment, drought stress inhibited the primary root growth, and with the increasing degree of drought stress, the primary root length was significantly lower than that of the normal-watering group. The spraying of SL significantly promoted the growth of primary roots, and the difference was significant compared with the D group (*P* < 0.05). In particular, T2 had the most significant relief effect. Similar results have been found for SL in *A. thaliana*. Ruyter-Spira et al. [60] showed that SL positively regulates the primary root length in *A. thaliana*, and SL controls root elongation by regulating the content of *A. thaliana* growth hormone. The exogenous application of SL restores the root phenotype, increases the length of the original roots, and promotes the growth and development of plants under stress conditions.

Plants must constantly adjust their ABA levels to adapt to changes under physiological and environmental conditions. ABA accumulates rapidly under saline and drought conditions and is considered to be a “stress hormone” [61, 62]. The interactions among SL, growth hormone, ABA, and other plant hormones jointly regulate abiotic stresses. In particular, the interaction between SL and ABA is the key to abiotic stress tolerance [63, 64]. The interaction between SL and ABA plays an important role in integrating stress signals and regulating stomatal development and function. The results of this experimental study indicate that different concentration treatments and different time periods after hormone treatment exert different effects on the ABA content of *P. purpureum*. More ABA was produced *P. purpureum* leaves in the first 48 h after spraying SL. ABA may not yet be produced in large quantities during mild drought, and SL application may have stimulated ABA production and maintained a dynamic balance between the hormones to enhance plant tolerance. With increased drought severity, the ABA content of the D group increased continuously, whereas the application of SL alleviated the adverse effects of drought, such that the ABA content did not increase further and decreased significantly compared with the D group. The genes related to the SL signaling pathway may have also co-regulated ABA levels [65, 66]. Toh et al. [67] showed that the exogenous application of SL reduced the ABA content in *A. thaliana* seeds and reduced dormancy induced by heat stress. A recent study in soybean seeds also showed that SL application reduced ABA levels in soybean seeds under alkali stress and promoted seed germination [68].

PEPC, NADP-ME, and PPDK are key enzymes of C_4_ photosynthesis and their main role is to concentrate CO_2_ for the Calvin cycle. Under adverse conditions, the photosynthesis efficiency of plants can be enhanced, stress tolerance improved, and crop yield ultimately increased by regulating the activities of key photosynthesis enzymes in plants [69–73]. In the present experiment, PEPC and PPDK activities increased under drought stress, suggesting that *P. purpureum* can cope with drought by elevating the activities of key enzymes of leaf photosynthesis, consistent with the findings of Doubnerová et al. [74]. However, NADP-ME activity decreased under drought stress, and enzyme activity may have been reduced during the NADP-ME-catalyzed production of pyruvate, NADPH, and CO_2_. PEPC, PPDK, and NADP-ME activities increased after spraying with SL, and the difference was significant (*P* < 0.05) compared with the D group. This finding indicated that the ability of *P. purpureum* to fix CO_2_ was significantly enhanced and photosynthesis performance was significantly improved after SL application. The NADP-ME activity in D, T1, T2, and T3 and the PEPC and PPDK activities in D, T2, and T3 were positively correlated with the net Pn of the leaves, indicating that the key enzymes of the photosynthesis photosynthesis pathway played important roles in their photosynthesis.

## Conclusions

In conclusion, the exogenous application of SL under drought stress significantly increased photosynthetic performance of *P. purpureum* leaves, reducing leaf photochemical damage, improving light-energy-utilization efficiency, and thus improving its adaptability in drought environment. Furthermore, SL application also increased the accumulation of biomass and the length of primary roots of *P. purpureum* to promote the growth of *P. purpureum* under drought conditions for better resistance to drought.

## Methods

### Test materials

The test material was *P. purpureum*, which was collected from the nursery of the National Juncao Engineering Technology Research Center of Fujian Agriculture and Forestry University. SL was purchased from Beijing Solabao Technology Co.

### Experimental design

The experiments were performed in a greenhouse, and *P. purpureum* was cultivated using the soil-cultivation method. When *P. purpureum* grew to seven leaves, *P. purpureum* was selected and divided into six treatment groups: control group (N), with normal-watering treatment and SL concentration of 0; drought-stress group (D), with watering stopped for 7 days to induce natural drought, soil moisture content maintained at 40–45%, and SL concentration of 0; and T1, T2, T3, and T4, sprayed with 1, 3, 5, and 7 μmol·L^− 1^ SL after drought stress, respectively. Five replicates were set up for each treatment. In the SL-treatment group, each SL concentration was sprayed onto *P. purpureum* leaves at 8:00 every morning continuously for 2 days on the basis that the leaves and the back of the leaves were covered with water droplets. Photosynthesis indices and Chl fluorescence were measured at 24, 48, and 120 h after SL treatment. The root length and fresh dry weight of leaves and roots were measured. Leaf and root samples were collected, and the samples were rapidly transferred to a − 80 °C refrigerator for storage after quick freezing with liquid nitrogen.

### Determination of photosynthesis parameters

The photosynthesis parameters of *P. purpureum* were measured using a CIRAS-3 portable photosynthesizer (PP-Systems Company Amesbury, MA01913, USA). The light intensity was set at 1200 μmol·(m^2^·s)^− 1^, the CO_2_ concentration was set at 380 μmol·mol^− 1^, and the air relative humidity was set at 75%. The photosynthesis parameters were measured under these conditions. The net Pn [μmol·(m^2^·s)^− 1^], Gs [μmol·(m^2^·s)^− 1^], Tr [mmol·(m^2^·s)^− 1^], Ci (μmol·mol^− 1^), and water-use efficiency (WUE; mmol·mol^− 1^) were measured in each treatment group. Five plants were measured for each treatment.

### Chl fluorescence determination

The maximum photochemical efficiency (Fv/Fm) and the performance index based on absorbed light energy (PIabs) were determined using a plant-efficiency analyzer. The top third unfolded leaf of the plant was selected, and the leaf was dark-adapted for 20 min before Chl fluorescence-parameter determination. Five plants were measured for each treatment.

### Biomass determination

*P. purpureum* was rinsed with distilled water, and the leaves and roots were dried and cut. The fresh weight of *P. purpureum* was immediately weighed with an analytical balance. Then, the plant was dried to constant weight with an electric blast dryer at 105 °C, and its dry weight was obtained. Three plants were measured per treatment. The formula for calculating the water content was as follows: water content (%) = (fresh weight − dry weight) / (fresh weight) × 100.

### Root-length determination

Three plants were taken from each treatment at 24, 48, and 120 h after SL treatment, and the length of the main root of *P. purpureum* was measured in centimeters using a meter ruler (minimum scale of 1 mm).

### Determination of ABA content

Leaf and root samples were taken at 24, 48, and 120 h after SL treatment. Then, they were snap frozen in liquid nitrogen and transferred into a − 80 °C refrigerator. The leaf and root samples were ground into powder with liquid nitrogen, and 0.1 g of sample was loaded into a centrifuge tube. Then, 0.9 mL of PBS buffer was added, shaken well, centrifuged at 3000 g and 4 °C for 10 min, and prepared for use. Ten microliters of supernatant was collected for ABA content determination according to the instructions of a Phytohormone Abscisic Acid (ABA) ELISA Kit (YX-010201P, purchased from Shanghai Preferred Biotechnology Co.). Three plants were measured for each treatment.

### Determination of key enzyme activities of photosynthesis pathways

Leaf sampling was performed at 48 h after SL treatment. The samples were snap frozen in liquid nitrogen and transferred into a − 80 °C refrigerator. The leaf samples were ground into powder with liquid nitrogen, and 0.1 g of the sample was loaded into a centrifuge tube. After adding 1 mL of extraction solution, it was homogenized in an ice bath, centrifuged at 8000 g and 4 °C for 10 min, and placed on ice. Ten microliters of supernatant was collected to determine the key photosynthesis enzyme activities according to the instructions of a NADP-ME kit (YX-W-A104, purchased from Shanghai Preferred Biotechnology Co.), a PEPC kit (YX-W-B203, purchased from Shanghai Preferred Biotechnology Co.), and a PPDK kit (YX-W-PPDK, purchased from Shanghai Preferred Biotechnology Co.). Three plants were measured for each treatment.

### Statistical analysis

Data were statistically analyzed using SPSS 20.0 (SPSS Inc., Chicago, IL, USA). One-way ANOVA and Duncan’s multiple comparison analysis were used to test the significance of differences between treatments (*P* < 0.05). The analysis results were expressed as the mean and standard error.

## Data Availability

All data generated or analyzed during this study are included in this published article.

## Abbreviations

SL: Strigolactone
ABA: Abscisic acid
WT: Wild-type
Chl: Chlorophyll
ROS: Reactive oxygen species
Pn: Net photosynthetic rate
Gs: Stomatal conductance
Tr: Transpiration rate
Ci: Intercellular CO_2_ concentration
WUE: Water use efficiency
Fv/Fm: Maximum photochemical efficiency
PIabs: Performance index based on absorbed light energy
CAM: Crassulacean acid metabolism
PEPC: Phosphoenolpyruvate carboxylase
PPDK: Pyruvate phosphate dikinase
NADP-ME: NADP-malic enzyme
